# Modeling Global Genomic Instability in Chronic Myeloid Leukemia (CML) Using Patient-Derived Induced Pluripotent Stem Cells (iPSCs)

**DOI:** 10.3390/cancers15092594

**Published:** 2023-05-03

**Authors:** Gladys Telliam, Christophe Desterke, Jusuf Imeri, Radhia M’kacher, Noufissa Oudrhiri, Estelle Balducci, Micheline Fontaine-Arnoux, Hervé Acloque, Annelise Bennaceur-Griscelli, Ali G. Turhan

**Affiliations:** 1 INSERM UMR_S_1310, Université Paris Saclay, 94800 Villejuif, France; 2 Faculté de Médecine Paris Saclay, Université Paris Saclay, 94270 Le Kremlin-Bicêtre, France; 3 APHP Paris Saclay Service d’Oncohématologie Moléculaire et Cytogénétique Hôpital Paul Brousse, 94800 Villejuif, France; 4 APHP-Paris Saclay Service d’Hématologie-Bicêtre, 94270 Le Kremlin Bicêtre, France; 5 INGESTEM National iPSC Infrastructure, 94800 Villejuif, France; 6 Centre for iPSC Therapies (CITHERA) INSERM UMS 45, Génopole, 91100 Evry, France

**Keywords:** CML, iPSC, genomic instability

## Abstract

**Simple Summary:**

Cancers progress and become resistant to therapies by acquiring novel, unpredictable genomic events. Chronic myeloid leukemia (CML) is a blood cancer characterized by the progression from a chronic phase towards an aggressive acute leukemia called “blast crisis” due to the accumulation of genomic abnormalities in the genetically unstable leukemic clone. The aim of our work was to reproduce these events using induced pluripotent stem cells (iPSCs) harboring the Philadelphia chromosome. These iPSCs with unlimited proliferation potential can be used to generate large numbers of leukemic cells in vitro. We show here that we can also use them for inducing genomic instability by mutagenesis, giving rise to leukemic cells harboring genomic alterations found in a large cohort of patients in blast crisis. We thus show that this iPSC-based “blast crisis in a dish” technology could be used for gene discovery and drug targeting strategies in CML and other hematological malignancies.

**Abstract:**

Methods: We used a patient-specific induced pluripotent stem cell (iPSC) line treated with the mutagenic agent N-ethyl-N-nitrosourea (ENU). Genomic instability was validated using γ-H2AX and micronuclei assays and CGH array for genomic events. Results: An increased number of progenitors (x5-Fold), which proliferated in liquid cultures with a blast cell morphology, was observed in the mutagenized condition as compared to the unmutagenized one. CGH array performed for both conditions in two different time points reveals several cancer genes in the ENU-treated condition, some known to be altered in leukemia (BLM, IKZF1, NCOA2, ALK, EP300, ERG, MKL1, PHF6 and TET1). Transcriptome GEO-dataset GSE4170 allowed us to associate 125 of 249 of the aberrations that we detected in CML-iPSC with the CML progression genes already described during progression from chronic and AP to BC. Among these candidates, eleven of them have been described in CML and related to tyrosine kinase inhibitor resistance and genomic instability. Conclusions: These results demonstrated that we have generated, for the first time to our knowledge, an in vitro genetic instability model, reproducing genomic events described in patients with BC.

## 1. Introduction

Chronic myeloid leukemia (CML) is the prototype of a clonal malignancy of the hematopoietic stem cell [1]. The initiation of the disease by the appearance of the Ph1 chromosome in a very primitive hematopoietic stem cell is followed invariably by the progression of the clone, acquiring novel abnormalities leading to clonal progression, accelerated and blast phases of the disease [2]. This natural history has now been modified by the use of tyrosine kinase inhibitors (TKIs) as first-line therapies, ensuring—in patients with a deep molecular response—prolonged survival on therapy [3]. However, in the current TKI era, progression toward blast crisis (BC) still occurs in 3–5% of patients, potentially due to clonal selection by TKI therapies [4,5]. Although the great majority of CML cases are diagnosed in chronic phase, some patients may be diagnosed, albeit much rarely, in the accelerated phase or BC—defined by a differentiation arrest of leukemic progenitors and lower sensitivity to TKI. Indeed, in one of the largest cohort analyses, the overall survival of CML-BC patients was 12 months [6].

Blast crisis is characterized by the progressive occurrence of genomic abnormalities due to BCR::ABL1-associated genetic instability inherent to leukemic cells [7]. Seminal and now classical works have been established using serially collected patient samples, main cytogenetic and molecular events observed in patients during the progression of the disease towards accelerated and eventually blast phase. These include cytogenetic abnormalities identified as “major” and “minor” routes [8,9].

Several works performed using patient samples and models have also identified, during the last two decades, major molecular events involved in this progression, such as TP53 mutations [10]. BCR::ABL1-expressing cells are also prone to developing a mutator phenotype [11], which could be due to the presence of increased oxidative stress [12], which leads to clonal selection under the influence of TKI therapies [13]. CML cells are finally doomed by the deficiency of several DNA repair mechanisms involving DNA-PKcs [14] and BRCA1 [15], as well as abnormalities of specific DNA repair genes, such as Bloom [16], and other repair mechanisms, such as NER [17] and MMR [18]. The genetic instability of CML cells is also enhanced by reactive oxygen species (ROS) production, which increases the mutator phenotype and favors the occurrence of additional mutations, such as RUNX1, ASXL1 and IKZF1 [19,20].

iPSC technology offers the possibility of obtaining an unlimited number of pluripotent cells which, despite difficulties of inducing definitive hematopoiesis [21], can be used for modeling some aspects of hematopoiesis, as it is possible to generate terminally differentiated progenitors and differentiated cells.

The occurrence of these genomic events is difficult to predict in individual patients. CML patient-derived iPSCs express BCR::ABL1 [22,23], allowing the possibility of enhancing global abnormalities by in vitro mutagenesis. In order to explore the possibility of generating such a model from an iPSC derived from a CP-CML patient, we have chosen an in vitro mutagenesis approach using N-ethyl-N-nitrosourea (ENU). Here, we show that CML patient-derived iPSCs can be used to generate an experimental model mimicking cytological and genomic instability patterns observed in primary blast crisis cells. Moreover, we show that abnormalities induced by this technology are representative of those described in large databases of genomic events described in primary CML patients in blast crisis.

## 2. Materials and Methods

### 2.1. Derivation and Characterization of Patient-Derived iPSCs

Generation of CML-iPSC PB32 has been previously described [22]. Briefly, peripheral blood cells were obtained at diagnosis from a 14-year-old CML patient with informed consent according to the declaration of Helsinki. This patient was treated with Imatinib mesylate as a first-line therapy. At 12 months post-therapy, there was no major molecular response, and an allogenic bone marrow transplant was performed. The patient has remained in complete remission since the transplant in 2004. To generate iPSCs, cryopreserved CD34+ cells were used, as previously described [22]. All analyses were performed using a polyclonal stock of iPSC. Control cells used for these experiments included PB33, an iPSC cell line that was obtained from bone marrow CD34+ cells using Sendai virus. CML and control iPSC have been characterized for their pluripotency using cell surface markers as well as by generation of in vivo teratoma assays in immunodeficient mice [22].

### 2.2. iPSC Cultures

iPS cell lines were maintained on the mitomycin-C-inactivated mouse embryonic fibroblast feeder cells with DMEM-F12 supplemented with 0.1 mg/mL bFGF, with or without N-ethyl-N-nitrourea 10 µg/mL or 50 µg/mL and treated 2 h with 1 mg/mL Collagenase type IV (Gibco Thermofisher, Paris France by life technologies ref 17104-019).

### 2.3. Embryoid Body Assays

To induce embryoid body (EB) formation, iPS cells at day 6–7 after cell passage were treated with collagenase IV. Clumps were cultured in Iscove’s modified Dulbecco’s medium (IMDM, Invitrogen) supplemented with 1% penicillin/streptomycin, 1 mM L-glutamine, 15% fetal calf serum (FCS, Invitrogen, Paris, France), 450 μM monothioglycerol, 50 μg/mL ascorbic acid (Sigma Aldrich, Paris, France) and 200 μg/L transferrin (Sigma Aldrich Paris, France), and supplemented with hematopoietic cytokines: 100 ng/mL stem cell factor (SCF), 100 ng/mL fms-like tyrosine kinase 3 ligand (Flt-3L) and 50 ng/mL thrombopoietin (TPO) (all from Peprotech, Thermofisher, Paris France). ESC and iPSC-derived EB were cultured in ultra-low attachment 6-well plates (Costar, Paris, France) for 16 days. Media was changed two or three times depending on EB proliferation. All cultures were incubated at 37 °C in 5% CO_2_.

### 2.4. Blast-Colony Forming Assays

Blast colony-forming cell (Bl-CFC) assays were performed using iPS cell pellets resuspended in Stemline II Hematopoietic Stem Cell Expansion medium (Sigma-Aldrich S0192 Paris France) supplemented with 1% penicillin/streptomycin and L-glutamine, 50 ng/mL rhVEGF (Peprotech Thermofisher, Paris France) and 50 ng/mL rhBMP4 (Peprotech Thermofisher Paris, France). After 48 h, we added 20 ng/mL of rhSCF (Peprotech Thermofisher Paris, France), rhTPO (Miltenyi Biotec, Germany) and rhFLT3L (Peprotech Thermofisher Paris, France). The embryoid bodies at day 3.5 were collected, dissociated with pre-heated stable trypsin replacement enzyme TrypLE Express (Gibco by Life technologies ref 12605-10 Paris France) and filtered with 40 µm Nylon Mesh sterile cell strainer (Fisher Scientific Paris France). Cells were resuspended in Stemline II Hematopoietic Stem Cell Expansion medium (Sigma-Aldrich S019P2 Paris France) supplemented with 1% penicillin/streptomycin and L-glutamine, 50 ng/mL rhVEGF, rhBMP4, rhFLT3L and rhTPO, 20 ng/mL FGFb (Peprotech Thermofisher Paris, France) and 5 Units/mL EPO (Peprotech Thermofisher Paris, France) and transferred in Methocult SF H4436 (Stem cell technologies, Vancouver Canada) for 5 to 7 days.

### 2.5. Hematopoietic Differentiation

iPSC-derived clonogenic assays were performed either from EBs or BL-CFC. Cells were plated at 10 × 10^3^ cells/mL into MethoCult GF (H4435 StemCell Technologies Vancouver, Canada). Hematopoietic CFC was counted on day 19.

### 2.6. Western Blots

Cells were lysed in ice with RIPA buffer containing (NaCl (200 mM), Tris (pH 8; 50 mM), Nonidet P40 (1%), acid deoxycholate (0.5%), SDS (0.05%), EDTA (2 mM)) supplemented with 100 µM phenylmethylsulfonyl fluoride (PMSF), 1 mM sodium fluoride (NaF), 1 mM orthovanadate (Na_3_VO_4_). Separation of proteins occurred by electrophoretic migration on a 3–8% polyacrylamide gel under denaturing conditions. The proteins were then transferred in a semi-liquid condition on PVDF membrane pre-activated in methanol. After saturation with TBS Tween 5% BSA for 1 h, membranes were hybridized with primary (Anti-phosphoHistoneH2A.X (Ser139) clone JBW Millipore Paris France) and secondary antibodies coupled to HRP. They were then revealed by chemiluminescence with SuperSignal West Dura or Femto reagents and data acquired using G:BOX iChemi Chemiluminescence Image Capture system.

### 2.7. Evaluation of Hematopoietic Cell Phenotypes

On day 19 of cultures, the methylcellulose cultures were washed with PBS. Living cells were counted in trypan blue and stained with the following antibodies in PBS 4%BSA at 4 °C 45 min: CD45Pe-Vio770 (Miltenyi), CD34Vioblue (Miltenyi), CD41aPE (BD), CD43FITC (Miltenyi), CD31FITC (Beckman Coulter), CD235aAPC (BD), IL1-RAP APC (R&D), CD38PE (Miltenyi), CD71PE (BD), CD14FITC (Beckman Coulter), CD33APC (BD), CD133APC (BD), BB9PE (BD), SSEA1PE (BD). After 1 h, cells were washed and resuspended in PBS 4%BSA with viability staining reagent 1 µg/mL (7-AAD) 7-aminoactinomycin D (Sigma Aldrich). Stained cells were analyzed with a MACSQuant 10 (Miltenyi Biotec) flow cytometer and Flowjo analysis software.

### 2.8. ENU Experiments

N-Ethyl-N-nitroso-urea (ENU) is a well-known alkylating mutagenic agent used for mutagenesis screens in both murine and human cells. In addition to its alkylating potential, it has been reported to induce nucleotide transitions generating large-scale mutations. CML-iPSC and control iPS cells were cultured in the presence or in the absence of ENU at the concentration of 10 μg/mL with daily addition of ENU as described above. Experiments were performed using iPSC cultured in ENU for either 40 days or 61 days. CGH array experiments were performed using EB’s derived hematopoietic cells and Bl-CFC generated from ENU-treated cultures as compared to cultures not treated with ENU.

For kinetic analysis, we recovered iPSC colonies on mouse embryonic fibroblasts (MEF). The “clumps” of colonies were incubated in DMEM-F12 medium supplemented with 50 µg/mL ENU at 37 °C for 2, 10 and 30 min. After treatment, the protein pellet was extracted from each condition in order to perform a kinetic analysis of the level of phospho γ-H2AX by Western blot.

### 2.9. Cytogenetic Analyses

Karyotype analyses were performed using cell pellets collected at different time points using standard methods, as previously described [22].

### 2.10. Micronuclei Analyses

The presence of micronuclei and anaphase bridges before and after exposure to ENU were analyzed, as described previously [24]. This methodology has also been widely used to determine genetic instability in leukemia [25]. Briefly, cells were incubated in a humidified atmosphere of 5% (*v*/*v*) CO_2_ in air at 37 °C until arrest and cell spread. The culture medium was discarded, and hypotonic shock was induced by incubating the cells with 18 mM KCl at room temperature. The cells were then fixed with acetic acid/ethanol (1:3, *v*/*v*), and the cell suspension was dropped onto the slides. The slides were stored at −20 °C until further use. Telomere and centromere staining was performed in order to detect the nature of micronuclei: with only telomere staining resulting mainly from acentric chromosomes or with telomere and centromere staining, which predominantly contained lagging chromosomes.

Automatic scoring of MN was performed using MNScore software (version 3.8.101 MetaSystems, Althaussen, Germany) with a Metafer 4 image analyzer (MetaSystems, Althaussen, Germany) comprised of a Zeiss Axioplan 2 imager to detect MN. An operator validated and excluded the false MN in cells. For each sample, 1000 cells were scored.

### 2.11. DNA Extraction

iPSCs, EBs and Bl-CFCs obtained from either ENU-treated or not treated CML-iPSCs, as well as from control iPSCs, were used for DNA extraction using standard methods with DNeasy Kit (Qiagen).

### 2.12. Array Comparative Genomic Hybridization

To perform CGH arrays, we used genomic DNA from ENU-treated iPSC-derived EBs and Bl-CFCs compared to CML-iPSCs without ENU treatment. We followed Agilent Oligonucleotide Array-Based CGH for Genomic DNA Analysis protocol. Briefly, we performed digestion from 500 ng of DNA with SureTag DNA labeling kit (4-packs). After DNA amplification, CML-iPSC was labeled with Cyanine 3 and the tested samples with Cyanine 5. Assembled chambers were loaded into the oven rotator rack; hybridizations were performed at 67 °C for 24 h at 20 rpm.

### 2.13. Transcriptome Dataset

Rosetta/Merck Human 25k v2.2.1 microarray Array data matrix of normalized log ratios from GSE4170 was downloaded on Gene Expression Omnibus website: (http://www.ncbi.nlm.nih.gov/geo/query/acc.cgi?acc=GSE4170 (accessed on 27 March 2017) and annotated with GPL2029 (http://www.ncbi.nlm.nih.gov/geo/query/acc.cgi?acc=GPL2029 (accessed on 27 March 2017) In this dataset, CD34+ cell samples belonging to the 3 experimental groups corresponding to the different evolution phases of chronic myeloid leukemia were analyzed: chronic phase, accelerated phase and blastic crisis [26].

### 2.14. Bioinformatics

Boxplot and two-sided Student’s *t*-test with Welch correction were performed in R Software version 3.2.3. Heatplot was generated with made4 package [27]. Functional enrichment was performed with Go-Elite Standalone software version 1.2 on the Gene Ontology Biological Process, KEGG and CommonsPathway databases included in Homo sapiens EnsMart77Plus (Ensembl—Biomart) update [28]. Functional interaction networks were performed with Cytoscape software version 3.2.1 [29]. Unsupervised principal component analysis was performed on gene expression profile with R package FactoMiner; *p*-value was calculated by group discrimination on first principal component axis. Pubmed gene prioritization was realized with gene valorization web application [30]. Circosplot on contingency table of Pubmed gene prioritization was realized with Circlize R package [31]. Circosplot of genomic aberrations with HG19 coordinates were performed with OmicCircos R package [32]. ROC curves were performed with ROCR R package. Genomic aberration gene candidates were also matched with important databases such as: database PLURINET for genes implicated in pluripotency [33], cancer genes COSMIC database: Catalogue of somatic mutations in Cancer (http://cancer.sanger.ac.uk/census (accessed on 27 April 2023)) [34] and transcription factors [35].

## 3. Results

### 3.1. Long-Term ENU Exposure Induces an Enhancement of CML-iPSC-Derived Hematopoiesis

**Figure 1:**
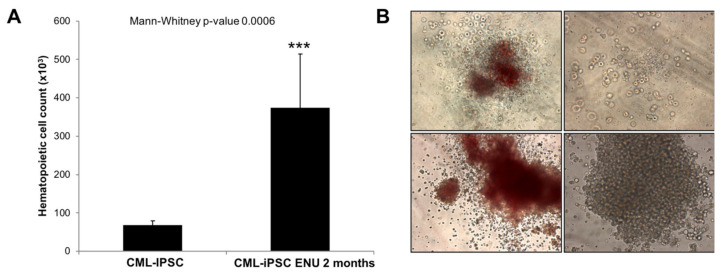
**Increased hematopoietic potential in CML-iPSCs under ENU exposure.** (**A**) Hematopoietic progenitor count from CML iPSCs before or after exposure to 10 μg/mL ENU during 2 months. A Mann–Whitney test was performed with PRISM software. (**B**) CFCs generated in methylcellulose cultures reveal large colonies with myeloid differentiation features as well as blast colonies (Magnification ×20). ***: Statistical significance.

To this purpose, we compared EB or Bl-CFC and hematopoietic cells from CFC assay derived from IPSC cultured under normal culture conditions or after ENU exposure (Figure 1A,B). As we can see in Figure 1B, after 2 months of ENU exposure, CML-iPSCs showed a significant increase in hematopoietic cell potential (Mann–Whitney *p* = 0.0006) (Figure 1A).

**Figure 2:**
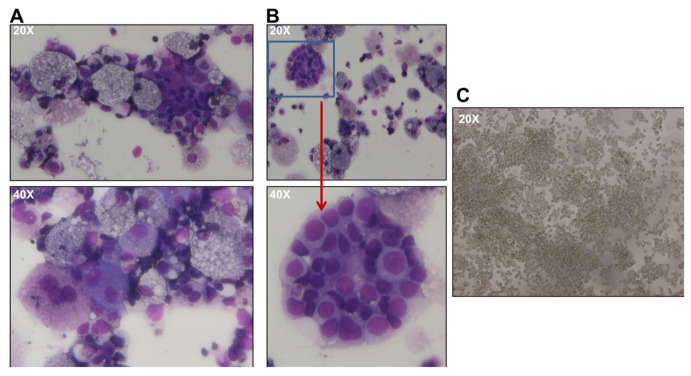
**May–Grunewald staining of hematopoietic colonies.** (**A**) Evidence of myeloid differentiation along with (**B**) blast colonies. (**C**) Proliferation of blast cells in liquid cultures in the presence of SCF, TPO and Flt3-ligand for >2 weeks.

Interestingly, in CFC assays generated from PB32-ENU cells, we observed larger colonies that proliferated in methylcellulose cultures (Figure 1B) as well as in liquid cultures in the presence of hematopoietic growth factors (SCF, TPO, Flt3L). Cytological features of these cells using May–Grünwald stain revealed the presence of myeloid cells with precursor and mature (myelocytes, metamyelocytes, neutrophils, monocytes/macrophages) cells as well as cells with undifferentiated blast cell morphology reminiscent of blast crisis cells (Figure 2A,B).

**Figure 3:**
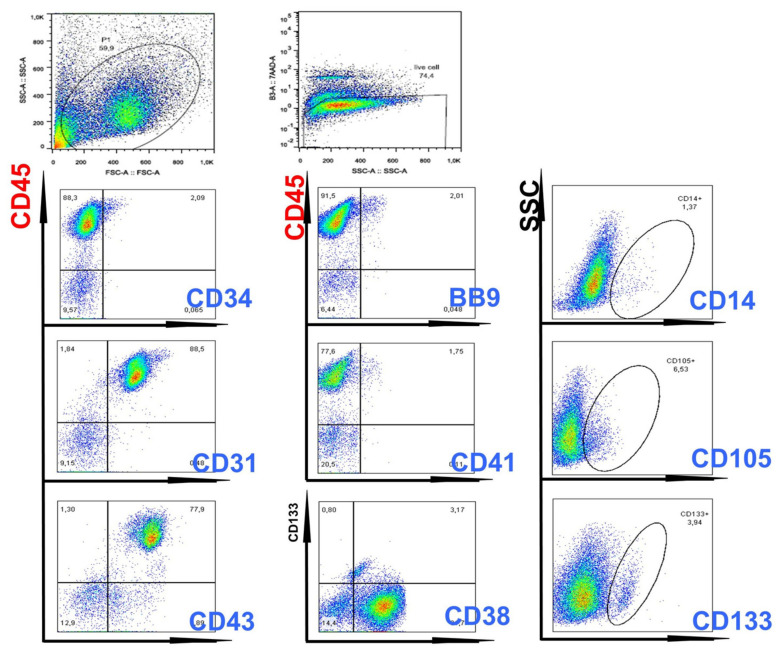
**Flow cytometry evaluation of hematopoietic cells generated from ENU-treated CML iPSCs**. As can be seen in this Figure, double-staining experiments showed the presence of CD45+/CD43+ and CD45+/CD38+ cells, demonstrating generation of hematopoietic cells with primitive characteristics.

FACS analysis showed that the majority of cells expressed CD45, with positivity of CD31, CD38, CD43 markers (Figure 3). Hemangioblast marker BB9, as well as CD133, CD26, SSEA1, CD13, CD33 and CD71-expressing cells, were also detected (Figure 3).

### 3.2. Genomic Instability of CML-iPSC Induced by BCR::ABL1 under ENU Exposure

Differences observed in the behavior of PB32 and PB32-ENU cells suggested that ENU could enhance DNA repair abnormalities known to be inherent to CML cells. We performed, for this purpose, a short-term exposure of our CML-iPSC to ENU and analyzed the kinetics of the accumulation of phospho-γH2AX in these cells.

**Figure 4:**
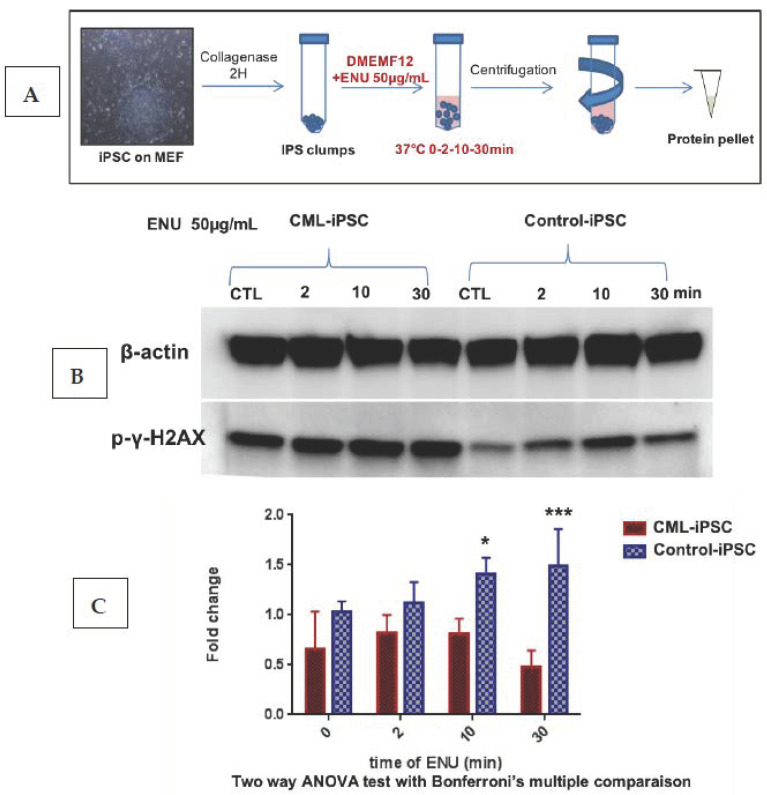
**Evaluation of the phosphorylation of** γ**-H2AX in the CML iPSC after short-time ENU exposure.** (**A**) CML iPSCs cultured on murine embryonic fibroblasts were collected after collagenase treatment, and the pellets subjected to a short time exposure to ENU followed by protein extractions. (**B**) Western blot analysis of the phosphorylation level of the histone variant gamma-H2AX (γH2AX) on Ser-139 after exposure of the CML-IPS cell line and control iPSCs to ENU at 50 μg/mL for 0, 2, 10 and 30 min (30 μg of protein per conditions). (**C**) Quantification of the γH2AX as compared to β-Actin with ImageJ software, histograms represent means of 3 independent experiments. A Two-way ANOVA test with multiple comparison and Bonferroni’s correction was made with PRISM software version 9. *, ***: Significant results.

We cultured iPSC colonies on mouse embryonic fibroblasts (MEF). The “clumps” of colonies were incubated with ENU at 37 °C for 2, 10 and 30 min. After treatment, the protein pellet was extracted from each condition in order to perform a kinetic analysis of the level of γH2AX (Figure 4A). Kinetic analysis of an iPSC control cell line revealed an increase of γH2AX expression level at 10 min of treatment with ENU with a progressive decrease over time. These observations suggest that ENU induced time-dependent DNA breaks and the DNA repair system is activated around 10 min after detection of the γH2AX signal. On the other hand, the CML-iPS cell line showed a gradual accumulation of γH2AX (Figure 4B) with a significant increase after 10 min and persistence of the phosphorylation after 30 min of ENU treatment (n = 3, Two-way ANOVA with Bonferroni’s correction) (Figure 4C). These results suggested the presence of a baseline increase of double-strand breaks in CML-iPSC, which persisted after ENU exposure, potentially due to a deficient DNA repair system.

The potential genomic instability of PB32-ENU cells was also evaluated by the micronucleus assays [24], which is a recognized test of the chromosomal instability of the eukaryotic cells. As can be seen in Supplementary Figure S1, the presence of spontaneous micronuclei was significantly higher in the mutagenized CML-iPSC lines (PB32-ENU) compared to their non-mutagenized counterparts and to the control cell line (PB33), validating the genomic instability of the mutagenized CML-iPSCs (Supplementary Figure S1).

We then wished to evaluate the presence of other features of cytogenetic and genomic abnormalities in PB32-ENU cells as compared to PB32 not treated with ENU. To this purpose, we performed cytogenetic analyses on CML-iPSCs after long-term exposure to ENU (PB32-ENU) compared to PB32 cultured without ENU. As can be seen in Table 1, in addition to Ph1 chromosome (Supplementary Figure S2), several cytogenetic abnormalities were identified (Table 1). Among these abnormalities (64 mitosis analyzed), we have noted the deletion of der 9q (3 mitosis/64) (5%) and the deletion of Chromosome 21 (5 mitosis/64) (8%) (Supplementary Figure S3). Despite several passages, control iPS cells exposed to ENU (PB33) did not develop any detectable cytogenetic changes (Supplementary Figure S3). To determine the genomic events at the molecular level, we performed CGH array experiments by comparing EB-derived CFC and Bl-CFC DNA generated from PB32-ENU as compared to PB32 cultured without ENU.

### 3.3. CGH Array Analysis of PB32-ENU-Derived Hematopoietic Cells

**Figure 5:**
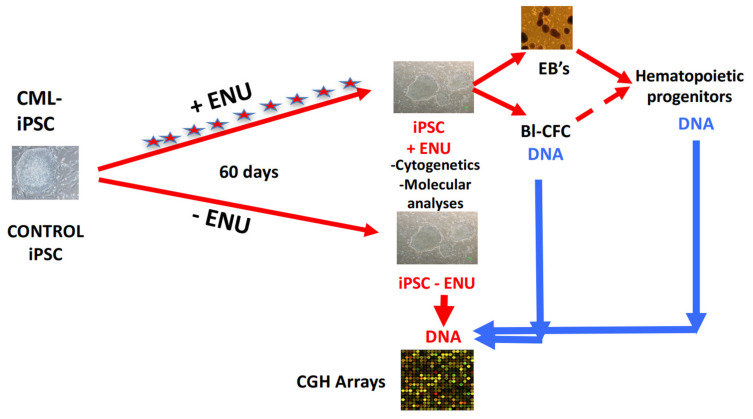
**Experimental protocol used for aCGH experimental procedure.** CML and control iPSCs were cultured in murine embryonic fibroblast layers in the presence or absence of ENU for 60 days, with daily medium changes containing or not ENU. On day +60, DNA extracted from hematopoietic progenitors generated via EBs or Blast-CFCs were used in CGH array experiments using DNA extracted from iPS cells without ENU exposure.

**Figure 6:**
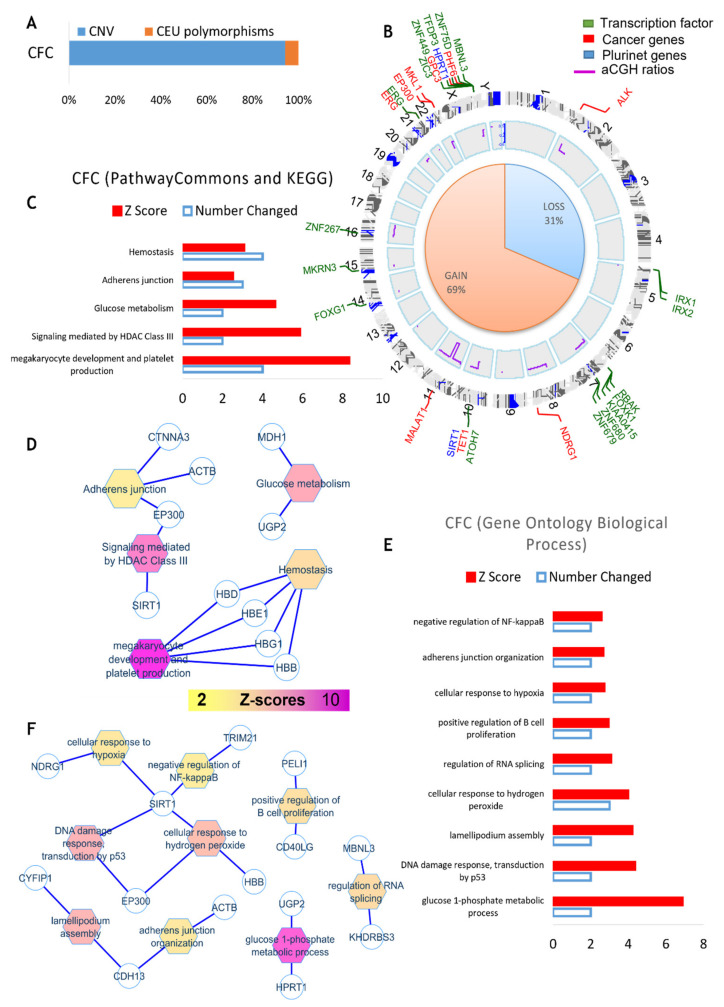
**aCGH analysis of EB-derived CFC cells.** (**A**) Quantitative genomics aberrations: copy number variations (CNVs). (**B**) Matching of genomic aberrations with transcription factor database, cancer gene database and pluripotency gene database circosplot, shown in green: 5 zinc finger ZNF genes and 2 IRX, in blue: pluripotency genes, in red: cancer genes. (**C**,**D**) Functional enrichment of genomic alterations of CFC on KEGG database. (**E**,**F**) Functional enrichment on Gene Ontology database.

Figure 5 shows the experimental strategy to perform this analysis. As can be seen in this figure, DNA from either PB32-ENU Embryoid body-derived hematopoietic colony-forming cells (CFC) or from blast-colonies (Bl-CFC) were hybridized with non-mutated iPSC PB32 DNA (Figure 5). Cytogenomics software from Agilent technologies with mosaic workflow allowed us to detect 20 quantitative genomics aberrations: copy number variations (CNVs), which comprised 313 gene loci. After filtration on the European Caucasian genomic polymorphism database, still, 295 gene loci were comprised in these genomic aberrations (Figure 6A and Supplementary Table S1). The majority of the gene loci events were affected by a gain of genomic DNA (69%) versus a loss (31%) (Figure 6B and Supplementary Table S1). Matching these genomic aberrations with the transcription factor database, cancer gene database and pluripotency gene database allowed us to observe that these important deregulated actors are principally affected genes from chromosomes 5, 7, 21, 22, and X (Figure 6B). The majority of these genomic events have been found to be transcription factors such as zinc finger ZNF and IRX (Figure 6B). Some pluripotency genes were also identified, as well as cancer genes, such as TET1, ALK, EP300, ERG, MKL1 and PHF6, already described as being involved in leukemia. Functional enrichment of genomic alterations of EB-derived CFCs allowed us to highlight perturbations in megakaryocytic development with HBD, HBE1, HBG1 and HBB molecules and HDAC enzymes, including EP300 and SIRT1 (Figure 6C,D). Functional enrichment on the Gene Ontology database allowed us to identify SIRT1, EP300 and CDH13 as key genes, especially SIRT1, which is implicated in p53-dependent DNA damage in response to hydrogen peroxide and cellular response to hypoxia. SIRT1 is also involved in the negative regulation of NF-kappaB (Figure 6E,F).

**Figure 7:**
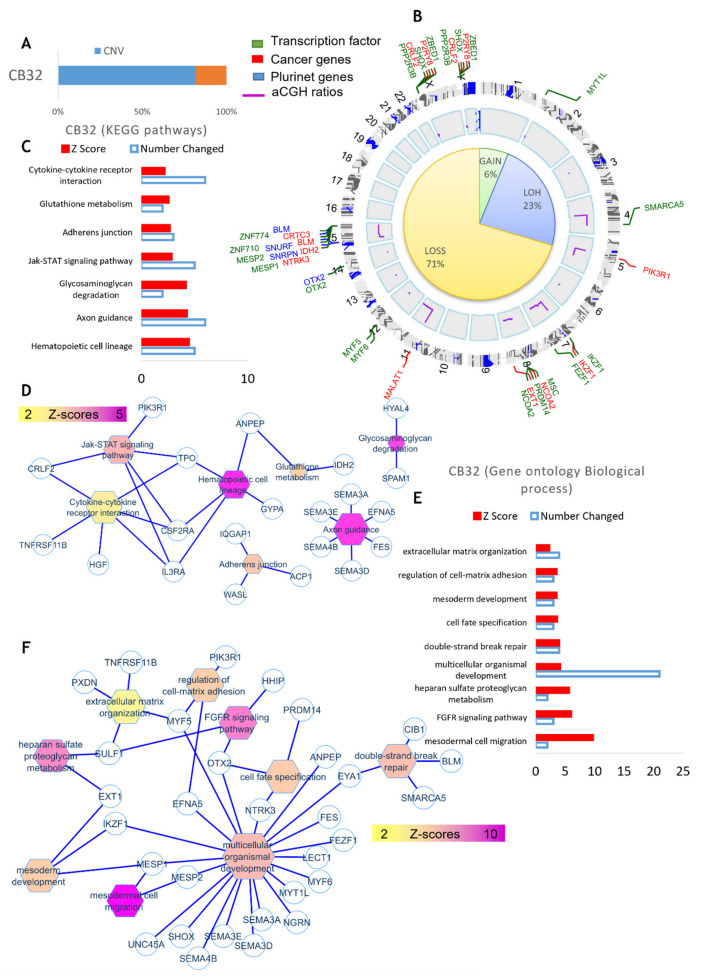
**aCGH analysis of Bl-CFC-progenitor cells.** (**A**) Cytogenomics software from Agilent technologies with mosaic workflow: quantitative genomics aberrations (copy number variations CNVs which comprised 332 gene locus). After filtration on European Caucasian genomic polymorphism database, 255 gene loci were comprised in these genomic aberrations. (**B**) Percentage of genomic loss, gains and LOH (loss of heterozygosity), match of genomic aberrations with transcription factor database, cancer gene database and pluripotency gene database. (**C**,**D**) Functional enrichment of genomic alterations of Bl-CFC on KEGG database. (**E**,**F**) Functional enrichment on Gene Ontology database.

When the same analysis was performed using DNA of Bl-CFC, 22 quantitative genomics aberrations were detected. These included copy number variations (CNVs), which comprised 332 gene loci. After filtration on the European Caucasian genomic polymorphism database, still, 255 gene loci were found to be comprised in these genomic aberrations (Figure 7A and Supplementary Table S2). The majority of these events represented a loss of genomic DNA (71%) with a loss of heterozygosity (23%), and 6% of them represented gains of DNA (Figure 7B and Supplementary Table S2). Matching these genomic aberrations with the transcription factor database, cancer gene database and pluripotency gene database allowed us to demonstrate that these important deregulated actors were principally involved on chromosomes 7, 8, 15, Y and X (Figure 7B). Circosplot analysis also allowed us to show that the majority of these events involved transcription factors, such as MESP, implicated in mesodermal cell migration and IKZF1 (Figure 7B,F). Several genes that we identified included IDH2, NCOA2, IKZF1 and BLM described as being involved in leukemia (Figure 7B,F). Functional enrichment of genomic alterations of PB32 iPSC on the KEGG database allowed us to highlight perturbations in hematopoietic lineage and cytokine-receptor interaction, affecting TPO, CSF2RA, ILRA, PIK3RA and CRFL2 involved in the JAK-STAT signaling pathway (Figure 7C,D). Functional enrichment on the Gene Ontology database allowed us to uncover gene implications (Figure 7E,F) such as IKZF1 and MESP1.

### 3.4. Comparison of Genomic Aberrations Identified by CGH in CML-iPSC as Compared to Primary Leukemic Blast Crisis Gene Profiling

**Figure 8:**
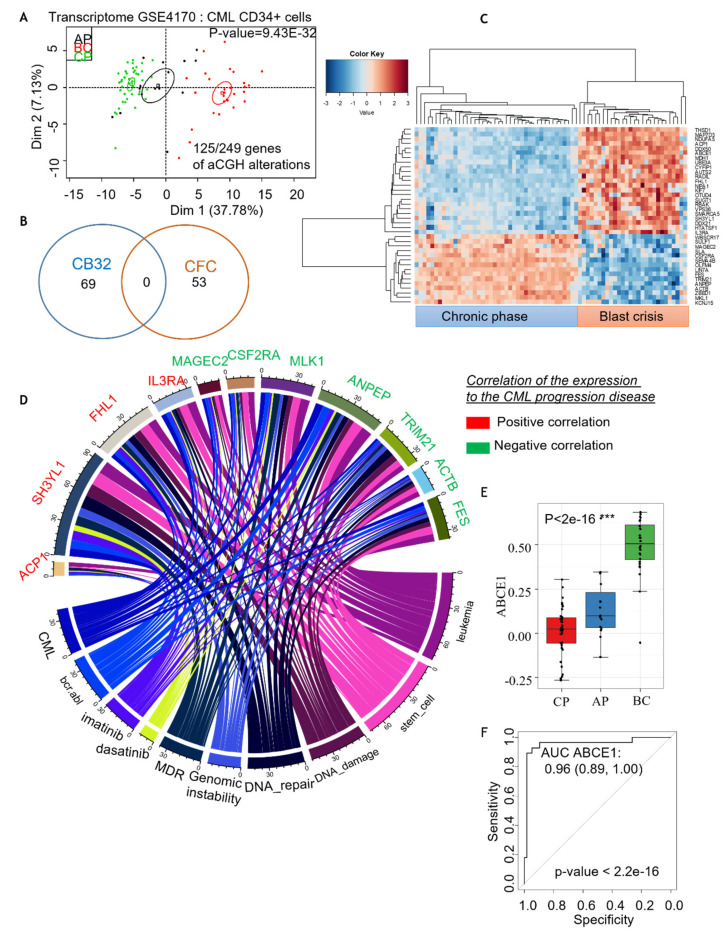
**Comparative analysis of genomic aberrations discovered in CML-iPSC aCGH with the gene** profiling described in primary CML CD34+ cells. (**A**) In silico analysis of genomic aberrations found in our study compared to gene profiling of CML CD34+ cells described in Radich [26] in the 3 different phases of disease: chronic phase (CP), accelerated phase (AP) and blast crisis (BC) were analyzed to extract information of genomic aberrations observed under ENU exposure, supervised with one-way ANOVA. (**B**) Genes related to CFC or Bl-CFC aCGH analysis. (**C**) Classification of the chronic phase samples compared to the blast crisis samples. (**D**) NCBI gene valorization performed one the best candidates correlated with progressive disease well known in CML pathophysiology. In green: predictive candidates to chronic phase; in red: predictive candidates to blast crisis. (**E**,**F**): Analysis of the expression of the ABCE1 gene expression between CP, AP and BC samples. ***: Statistical significance.

The transcriptomes of CML CD34+ cells in the three different phases of the disease were analyzed to extract information on genomic aberrations observed in CML PB32-ENU_derived hematopoietic progenitors. On 249 candidate genes, a supervised one-way ANOVA analysis was performed between the three phases of the disease (with 1000 permutations and *p*-value threshold less than 1e^−3^). This analysis identified 125 genes, and an unsupervised principal component analysis allowed us to obtain a very significant group discrimination (*p*-value of discrimination 9.43e^−32^; Figure 8A and Supplementary Table S3). Interestingly, some of these genes were identified in EB-derived CFCs and some in Bl-CFC-derived cells (Figure 8B). Unsupervised classification performed with the gene candidates mostly correlated with the progression of the disease, allowing a perfect reclassification of the chronic phase samples as compared to the blast crisis samples (Figure 8C). NCBI gene valorization performed on these best candidates, correlated with the progression of disease, identified eleven genes (Figure 8D) well known in CML pathology, genomic instability and tyrosine kinase inhibitor-related keywords in the published CML literature (Figure 8D). The majority of these candidates, including a group of 7 genes (7 of 11), are predictive for the chronic phase (Figure 8D), whereas the other group is predictive for blast crisis (4 of 11) (Figure 8D). Among the candidate genes predictive of blast crisis not reported in CML pathophysiology were the DDX family, including DDX50 and DDX21.

## 4. Discussion

In the current era of TKI therapies, the progression toward blast crisis has become a rare event, but it still occurs after several years and correlates with resistance to TKIs. Historically, experimental studies of advanced CML used cell lines derived from patients in blast crisis. The first of these cell lines was K562, largely used worldwide [36] but not very representative of a true BC sample, as the occurrence of additional oncogenic events during several passages over several years was highly probable [37].

Major cytogenetic events [8], as well as molecular events contributing to the differentiation arrest during BC, have been identified [10,38]. More recently, global genomic analyses performed in primary BC samples showed the enrichment of the CML genome by the occurrence of mutations, essentially of the polycomb repressive complex (PRC) [39]. However, these tools do not allow to study progressive changes occurring during the clonal evolution of the disease, which requires serial samplings.

Blast crisis in CML can also be modeled using murine CML models by using complementation assays using BCR::ABL1 and an oncogenic fusion protein such as NUP 98/HoxA9 [40,41]. In these murine models, the cooperation between BCR::ABL and the NUP98/HOXA9 gives rise to very aggressive leukemia in mice similar to acute leukemia [40,41]. However, the aggressivity of the models is not representative of human blast crisis.

Another strategy involves the overexpression of oncogenic fusion genes reported in human BC samples in CD34+ cells from CML with the goal of inducing a blast crisis in vitro. This strategy has been shown to be highly interesting by the lentivirus-mediated gene transfer of the NUP98/HOXA9 fusion gene in CML CP CD34+ cells, generating a transcriptional program reminiscent of the blast phase of the disease [42]. This strategy is of major interest but experimentally highly complex and dependent on several variables, including the secondary oncogenic driver genes chosen, in addition to BCR::ABL.

The power of the iPSC technology makes it possible to generate pluripotent stem cells from a leukemic blood sample with the ability to generate unlimited numbers of stem cells, which can then be induced to differentiate, as has been shown in AML [43]. In this work, we wished to apply this strategy with the further goal of evaluating the feasibility of generating a genetic instability model of CML using patient-specific-induced pluripotent stem cells. An iPSC cell line was established from a patient with TKI-resistant CML, as previously described [22]. These cells exhibited all features of pluripotency, including cell surface and in vivo teratoma-generating abilities [22], and have been cultured in a pluripotent state for several passages. We have shown that these cells express BCR::ABL1 and BCR::ABL1-signaling, which appears to be preserved during differentiation towards hematopoiesis generated via embryoid body or blast-cell colony assays [23]. Using clonogenic cell assays, we have shown that this iPSC cell line (PB32) exhibits an increased hematopoietic potential as compared to standard ES cells, such as H1 or control iPSC without BCR::ABL1 expression [22]. To mimic in vitro genetic instability, we have used ENU, a well-established mutagen and alkylating agent [44]. We have previously shown that ENU can be used in CML cell lines to generate ABL-kinase mutations that can be selected in vitro by TKI and allows in vitro production of clinically relevant mutations such as T315I substitution [45]. After testing the appropriate dosing experiments, we used ENU in vitro during each daily medium change. PB32-ENU cells were thus cultured for >60 days with PB32 counterparts cultured and passaged without ENU. This treatment did not appear to induce any toxicity, and no morphological changes in iPSC (data not shown). However, in hematopoiesis induction experiments, we observed a major increase in hematopoietic potential using either EB or Bl-CFC-derived hematopoietic cells (Figure 1). Hematopoietic cells generated in these cultures could be expanded in short-term cultures, revealing the presence of myeloid precursors and blast-like cells, reminiscent of an accelerated phase of CML (Figure 2), whereas such a pattern was not observed in iPSC cultured without ENU. Interestingly, cytogenetic analyses performed in PB32-ENU cells revealed the presence of several chromosomal abnormalities in addition to the Ph1 chromosome (Supplementary Figures S1 and S2). These included the presence, in several mitoses (3/64 analyzed), deletion of the derivative chromosome 9, which has been shown to occur at diagnosis [46] but also during progression toward blast crisis [47,48]. Another significant abnormality observed was the deletion of chromosome 21 in several mitoses in PB32-ENU cells (Table 1). None of these abnormalities are representative of a major or minor route, but they have been described in CML patients during blast crises [49]. The analyses of PB32-ENU cells as compared to PB32 also revealed the presence of extensive micronuclei formation, suggesting the induction of significant genomic instabilities in these cells (Supplementary Figure S1). Micronuclei are characteristics of cells undergoing DNA damage, especially in malignant cells [50]. They are formed during mitosis and can be due to the presence of double-strand DNA breaks [50]. Increased micronucleus frequency has been shown to be a feature of myelodysplastic syndromes and has been shown to be higher in patients with refractory anemia with excess blasts [51]. To analyze the chromosomal abnormalities at the genomic level, we performed CGH array analyses to compare PB32-ENU-derived hematopoietic cells or Bl-CFCs as compared to PB32 without ENU treatment (Figure 5). This analysis revealed several major genomic aberrations (Figure 6 and Figure 7). As can be seen in Figure 6, in hematopoietic cells, we have observed several losses (31%) or gains (69%), interestingly, in all chromosomes and some major genes involved in oncogenesis such as TET1, ALK, ERG and MALAT1 (Figure 6). Several transcription factor genes were also involved, such as ZNF or the ERG family of transcription factors (Figure 6). Functional enrichment on the Gene Ontology database showed the implication of SIRT1, EP300, CDH13 and SIRT1 being involved in TP53-dependent DNA damage.

The analysis performed using comparative hybridization of Bl-CFC DNA yielded additional information with losses (71%) and gains (6%), and matching these genomic aberrations with the transcription factor database, cancer gene database and pluripotency gene database allowed us to confirm that these essentially affect chromosomes 7, 8, 15, Y and X (Figure 7B). Circosplot analysis showed that the majority of these abnormalities implicate transcription factors such as MESP (implicated in mesodermal cell migration) and IKZF1 (Figure 7B,F). Amongst pluripotency genes, we have identified cancer genes (IDH2, NCOA2, IKZF1, BLM) which are already described as being involved in leukemia. Functional enrichment of genomic alterations of PB32 iPSC on the KEGG database highlights perturbations in hematopoietic lineage and cytokine-receptor interaction, affecting TPO, CSF2RA, ILRA, PIK3RA, CRFL2, cytokines and receptors, allowing activation of the JAK-STAT pathway (Figure 7C,D). Differences observed between genomic abnormalities in hematopoietic progenitor (CFC)-derived DNA and blast-cell colony (Bl-CFC)-derived DNA could be due to the occurrence of random genetic abnormalities induced by ENU throughout the genome.

We then asked whether the abnormalities identified using CGH arrays could be matched with genomic aberrations described in primary leukemic cells from blast crisis patients. Amongst 249 genes identified by aCGH in CML-iPSC, 125 were previously described in CML progression (Figure 8 and Supplementary Table S3). These positively correlating genes include IL3RA, ACP1, FHL1, SH3YL1 (Supplementary Figure S4). It has been shown that IL3RA (IL3 receptor alpha) is highly expressed in AML cells [52] and can be targeted using an anti-CD123 monoclonal antibody in CML blast cells [53]. Genes correlating negatively with CML progression included CSF2RA (GM-CSF alpha receptor), which is implicated in AML biology [54]. ANPEP is the gene coding for CD13 antigen, which has been shown to be associated with monocytic/myeloid differentiation [55]. It is of interest to note that we have identified this gene to be negatively correlated with CML progression. The relevance of some of the genes identified in the progression of CML in our screen will require further study.

## 5. Conclusions

In summary, in this work, we report the possibility of inducing genomic instability in CML patient-derived iPSCs with genomic alterations similar to those observed in a large cohort of “real-life” CML patients in blast crisis. Indeed, upon hematopoietic differentiation of these iPSCs, we were able to show cytological and genomic features of blast crisis in this patient-specific genomic background. The experimental methodology described here is unique as we show the possibility of generating a “blast crisis in the dish” model using an iPSC harboring the Philadelphia chromosome. The ability to induce an in vitro mutagenesis from leukemia-derived iPSCs could allow manufacturing of an unlimited number of blast cells and is highly attractive for gene discovery or drug screening purposes.

The limitations of the methodology are related essentially to the heterogeneity of the patient population and the efficiency and reproducibility of the mutagenesis strategy. In addition, given the fact that the iPSCs that we used in the work were derived from a patient with resistance to Imatinib, it remains to be determined if this in vitro mutagenesis strategy could be efficiently applied to other CML-patient-derived iPSCs.

Nevertheless, this first demonstration of the feasibility of using this approach to model blast crisis could open new perspectives for the future use of CML-iPSCs for disease modeling and, eventually, for uncovering novel targets. This model could also be used in other hematopoietic malignancies, such as myelodysplastic syndromes, with the goal of generating a genetic instability model that could allow experimental studies such as drug screening.

## Figures and Tables

**Table 1:** **Evaluation of Additional Chromosomal Abnormalities in iPSCs before and After ENU-induced mutagenesis.** PB32+ ENU: Ph1+ CML-IPSC line cultured with ENU. PB32 without ENU: Ph1+ CML-iPSC without ENU-mutagenesis. PB33 + ENU: Control Ph1-negative cell cultured with ENU. PB33-Ph1-: Control Ph1-negative cell line.

iPSC Line	# of Chromosome Involved	Mitosis Ratio	%
PB32 + ENU (Ph1+)	21	5 loss/64	8
	9	3 loss/64	5
	17	1 loss/64	>2
	19	1 loss/64	>2
	20	1 loss/64	>2
	13	1 loss/64	>2
	1	1 loss/64	>2
	2	1 loss/64	>2
PB32 without ENU (Ph1+)	None	/	/
PB33 + ENU (Ph1-neg)	None	/	/
PB33 without ENU (Ph1-neg)	None	/	/

## Data Availability

Not applicable.

## Supplementary Materials

The following supporting information can be downloaded at: https://www.mdpi.com/article/10.3390/cancers15092594/s1. Figure S1: Evaluation of additional chromosome abnormalities in CML-iPSC after long term ENU culture; Figure S2: Representative cytogenetic analyses of PB32 and PB33; Figure S3: Evaluation of global genomic instability by micronucleus assay in CML-IPSC, control iPSC and human embryonic stem cell line H1 cell line before and after ENU exposure; Figure S4: Genes with negative correlation to CML progression; Figure S5: Whole of western blot.
