# Supplementary material for: Modeling Global Genomic Instability in Chronic Myeloid Leukemia (CML) Using Patient-Derived Induced Pluripotent Stem Cells (iPSCs)
Source: Cancers (Basel). 2023 May 3;15(9):2594. doi: 10.3390/cancers15092594 (PMC10177163; doi:10.3390/cancers15092594)
Supplement: Supplementary file 1 [file cancers-15-02594-s001.zip › cancers-2251279-supplementary.pdf]

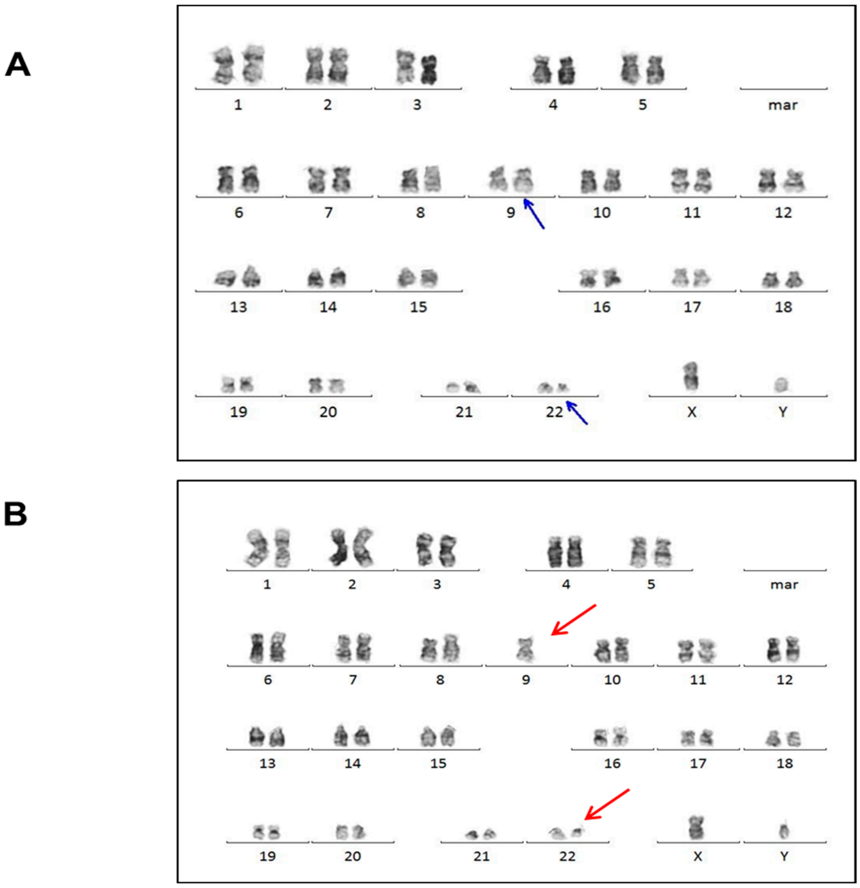

Supplementary Figure S1: **Evaluation of additional chromosome abnormalities in CML-iPSC after longterm ENU culture. (A)** Karyotype of CML IPSC with Ph1 chromosome and 9q+. **(B)** CML IPSC karyotype after 2 months of ENU exposure showing loss of Chromosome 9.

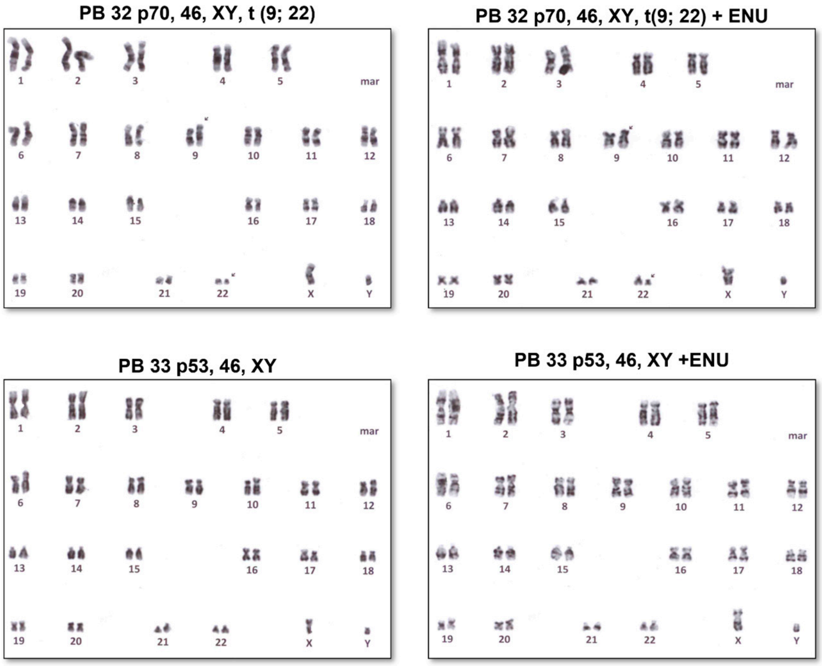

Supplementary Figure S2: Representative cytogenetic analyses of PB32 (CML-IPC) and PB33 (control iPSCshowing with and without long term ENU culture. P53: passage 53, P46: passage 46.

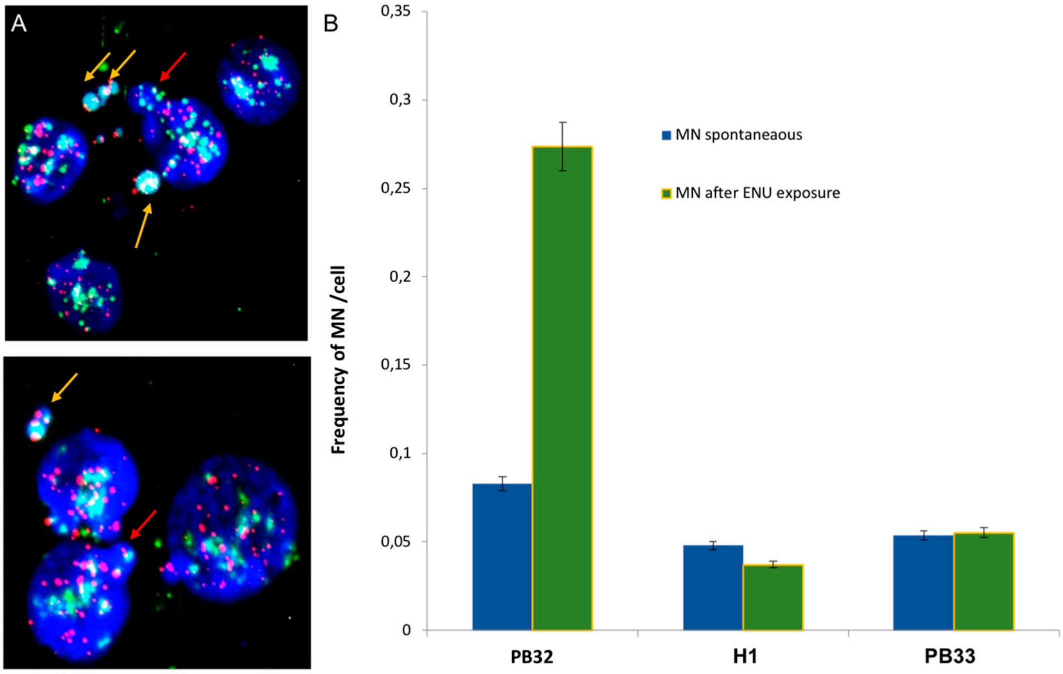

Supplementary Figure S3: Evaluation of global genomic instability by micronucleus assay in CML-IPSC,control iPSC and human embryonic stem cell line H1 cell line before and after ENU exposure. (A) Photograph of CML-IPS colonies after ENU exposure. Yellow Arrow show micronucleus and white arrow shows nucleus bud (NBUD's). **(B)** Micronucleus frequencies of CML-IPSC compared to control ESCand IPSC with and without long term ENU exposure.

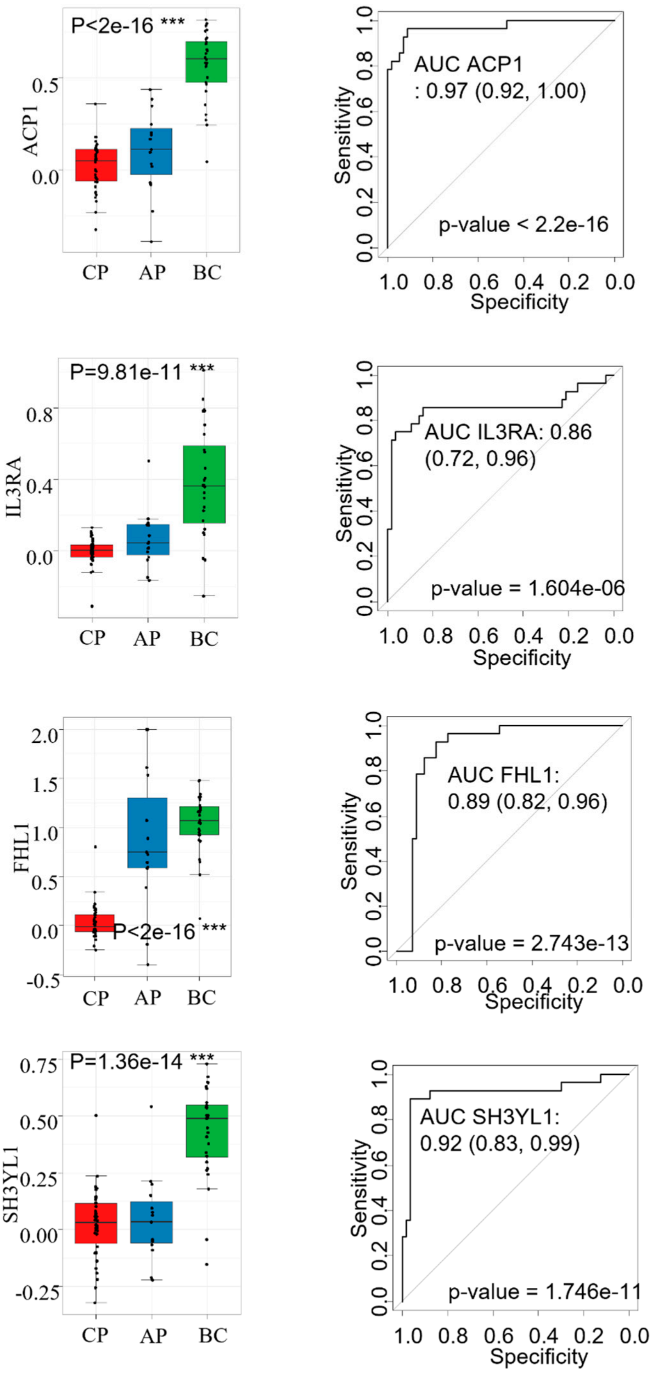

Supplementary Figure S4: Genes with negative correlation to CML progression.

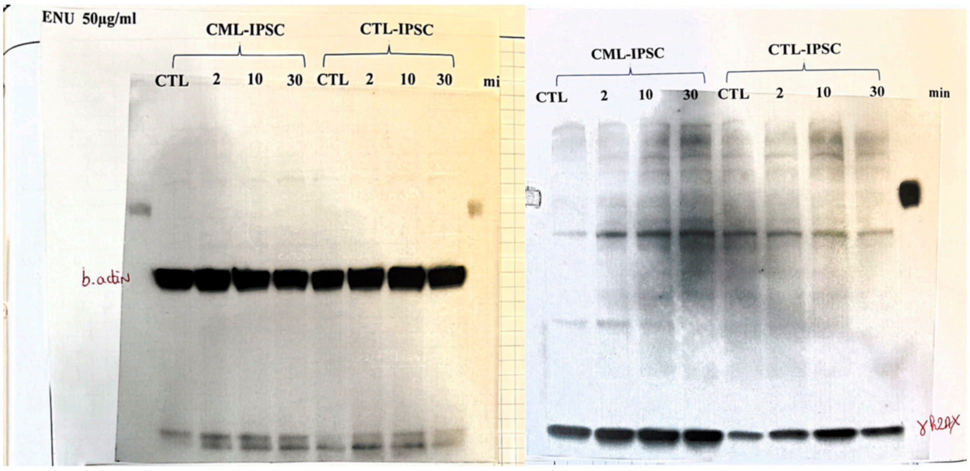

Supplementary Figure S5: Whole of western blot
